# Periprosthetic stress fracture around a well-fixed type 2B short uncemented stem

**DOI:** 10.1051/sicotj/2018031

**Published:** 2018-07-30

**Authors:** Pablo Ariel Slullitel, Jose Ignacio Oñativia, Lionel Llano, Fernando Comba, Gerardo Zanotti, Francisco Piccaluga, Martin Alejandro Buttaro

**Affiliations:** Hip Surgery Unit, Institute of Orthopaedics “Carlos E. Ottolenghi”, Italian Hospital of Buenos Aires, Buenos Aires Argentina

**Keywords:** Total hip arthroplasty, Short stem, Thigh pain, Stress fracture, Periprosthetic fracture

## Abstract

Despite the theoretical advantages of uncemented short stems, postoperative thigh pain is still matter of concern and can be attributed to different causes. We report a peculiar case of a stress fracture around a short cementless stem with cervico-metaphyseal fixation in an otherwise healthy patient. We implanted a MiniHip^TM^ stem in a 43 year-old male professional golf player for the treatment of primary osteoarthritis using a ceramic on ceramic bearing. Against medical advice, the patient started to play soccer at the 4th postoperative month and was completely asymptomatic to that extent; but at 8 months follow-up and without a history of trauma he started complaining about progressive hip pain. After ruling out infection and loosening, histological analysis from a bone biopsy confirmed the diagnosis of stress fracture. Although revision surgery was initially scheduled, pain started to decrease gradually with protected weight-bearing (crutches) and disappeared around the first postoperative year, remaining the patient asymptomatic at 2 and half years of follow-up, with radiographs depicting a healed fracture with a hypertrophic callus. We encourage surgeons to be aware of the existence of periprosthetic stress fractures as a source of thigh pain (sometimes intractable), and despite being infrequent, they should always be contemplated, providing that these cases can be managed conservatively with rest and limited weight-bearing. After this uncommon case, we suggest to align the stem in order to equally distribute loads onto the medial calcar and the lateral femoral cortical.

## Introduction

The main purposes of short stem designs are bone preservation, theoretical stress-shielding avoidance and ease in case of an eventual revision arthroplasty (due to loosening) in the future. However, whether short stems can achieve similar clinical and radiographic outcomes to that of standard length stems in the long-term and also maintain a low frequency of thigh pain is still a matter of controversy. Recent reports have emphasized on mild to excruciating thigh pain developing after short-stem total hip arthroplasty (THA) [1,2].

Thigh pain is a recognised cause of dissatisfaction at 1- and 2-year follow-up after uncemented THA, with a prevalence ranging from 3% to 25% [3]. It has been associated with larger stem sizes, high elasticity modulus of the stem and varus alignment; nonetheless, evidence remains controversial about the exact genesis of soreness in these cases [4,5].

Atraumatic periprosthetic stress fractures constitute an extremely unusual cause of femoral pain. Only a few reports have addressed this issue, especially around loose, cemented stems or modular ones [6–8]. To our knowledge, no cases of stress fractures had been reported around short stems in otherwise healthy patients. Thenceforth, we aimed to report a peculiar case of a stress fracture occurring around a type 2B, [9] short cementless stem with cervico-metaphyseal fixation (MiniHip™, Corin, Cirencester, United Kingdom).

## Case report

**Figure 1:**
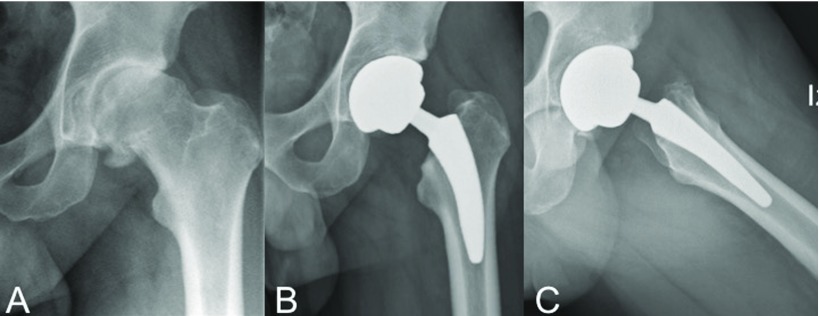
A Preoperative anteroposterior radiographs of a 43-yeard old male´s left hip with diagnosis of primary osteoarthritis B and C. Immediate postoperative anteroposterior (A) and lateral (B) radiographs showing adecuate implant positioning after implanting a MiniHip™ stem.

A 43-year-old professional golf player (Male, body mass index [BMI] 28.5) with unilateral Tönnis [10] grade 3 primary coxarthritis underwent an otherwise uneventful primary THA with a short cementless MiniHip™ stem (Corin, Cirencester, United Kingdom), as shown on Figure 1. Preoperative modified Harris Hip Score (mHHS) was 55 points [11]. A posterolateral approach with the patient positioned in lateral decubitus was done, under epidural hypotensive anaesthesia, with a single preoperative dose of intravenous tranexamic acid and cephazoline administration. Besides preoperative planning and in order to calculate limb lengthening, the Woolson method was used with a Steinman pin inserted proximal to the acetabulum, as a stable pelvic reference point [12]. Given the patient's level of activity, a fourth generation ceramic on ceramic (CoC) surface was selected (CeramTec™, Plochingen, Germany).

Postoperative rehabilitation protocol consisted of early weight-bearing as tolerated with crutches during the first 2 postoperative weeks, with fixed range of motion exercises at 90° of flexion, neutral internal rotation, 30° of external rotation and 45° of abduction for 3–6 weeks. Subsequently, progression to a single cane on the contralateral hand until the 21st postoperative day was indicated. Noncontact sports were allowed after the 3rd postoperative month and contact sports were allowed but not recommended after 6 months; nevertheless, the patient began to practice soccer at 4 months of follow-up.

The first 7 postoperative months developed in an uneventful fashion. However, around the 8th month of follow-up and without a history of trauma, the patient started complaining about progressive pain referred on the anterior thigh, more excruciating upon axial load. Defined by the patient as 8 points from the Visual Analogue Scale (VAS), pain could be only partially alleviated by non-steroidal anti-inflammatory drugs and protected weight-bearing.

**Figure 2:**
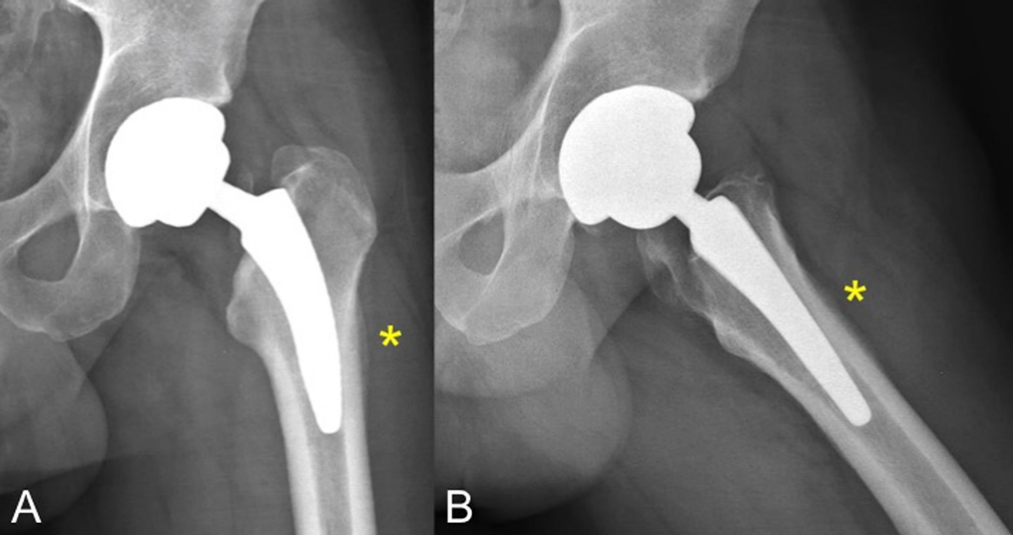
A and B. Anteroposterior (A) and lateral (B) x-ray views of the same patient's hip at 8 months of follow-up depicting bone remodelling and periosteal reaction located at the lateral and anterior femoral cortices (*), which correlated with unremitting pain that exacerbated with axial loading. No signs of prosthesis subsidence or loosening can be appreciated.

**Figure 3:**
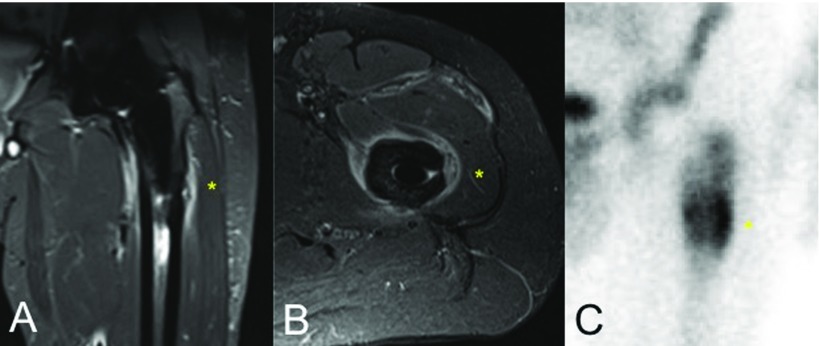
A and B. Fat-Sat magnetic resonance imaging sequences demonstrating intraosseous and periprosthetic bone oedema as well as soft-tissue inflammation at the coronal (A) and axial (B) views. C. Triple-phase bone scintigraphy showing high metaphyseal uptake surrounding femoral stem, especially at the lateral femoral cortical (*).

Radiographs evidenced a lateral cortical hypertrophy and periosteal reaction located at zones 2, 3, 8 and 9 of Gruen's classification in anteroposterior and lateral views, respectively, [13] without any signs of subsidence or loosening, as shown on Figure 2. Magnetic resonance imaging (MRI) revealed marked intraosseous oedema around the tip of the stem as well as around the periprosthetic subfascial soft tissues (Figures 3A and B). Additionally, bone scintigraphy displayed an increased bone turnover at the distal part of the lateral periprosthetic cortical within the three phases of tracer uptake, suggesting increased blood flow (in the arterial phase), tissue hyperaemia (second phase) and increased osteoblastic activity (third phase); which is compatible with a stress fracture [14] (Figure 3C). Infection was initially suspected but laboratory values were negative for any inflammatory reaction. Serum C-reactive protein value was 3 mg/ml whereas erythrocyte sedimentation rate was 8 mm/hour.

**Figure 4:**
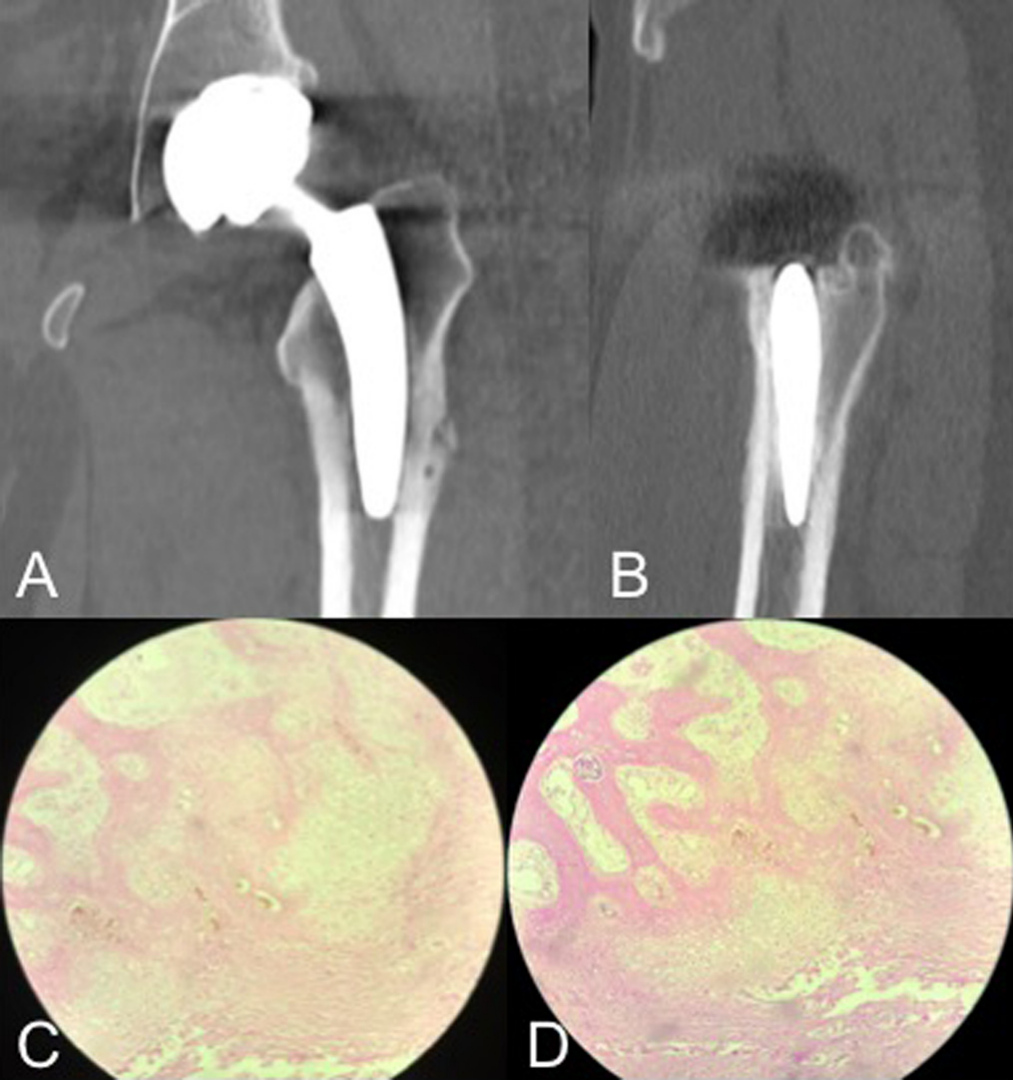
A and B. Coronal and sagittal computed tomography slices obtained at the time of bone biopsy, taken from the lateral femoral cortical at 9 months of follow-up depicting no evidence of stem loosening or sinking. C and D. 10x and 40x haematoxylin and eosin staining histological sections taken from a bone biopsy taken at the lateral femoral cortical of the patient's left hip at 9 months of follow-up. Pathology analysis reveals disorganized trabecular bone and cartilaginous tissue with peri-haversian bone formation areas and abundant capillaries (angiogenesis), as well as increased subperiosteal osteoblastic activity.

In this scenario, both an articular aspirate and a bone biopsy of the affected zone were performed at the operating room under general anaesthesia, in order to rule out periprosthetic infection and osteomyelitis. Culture results were negative for infection and pathology analysis revealed areas of osteoclastic resorption nearby haversian canals as well as areas of bone formation with abundant capillaries, increased subperiosteal osteoblastic activity and periosteal thickening (Figure 4) [15].

**Figure 5:**
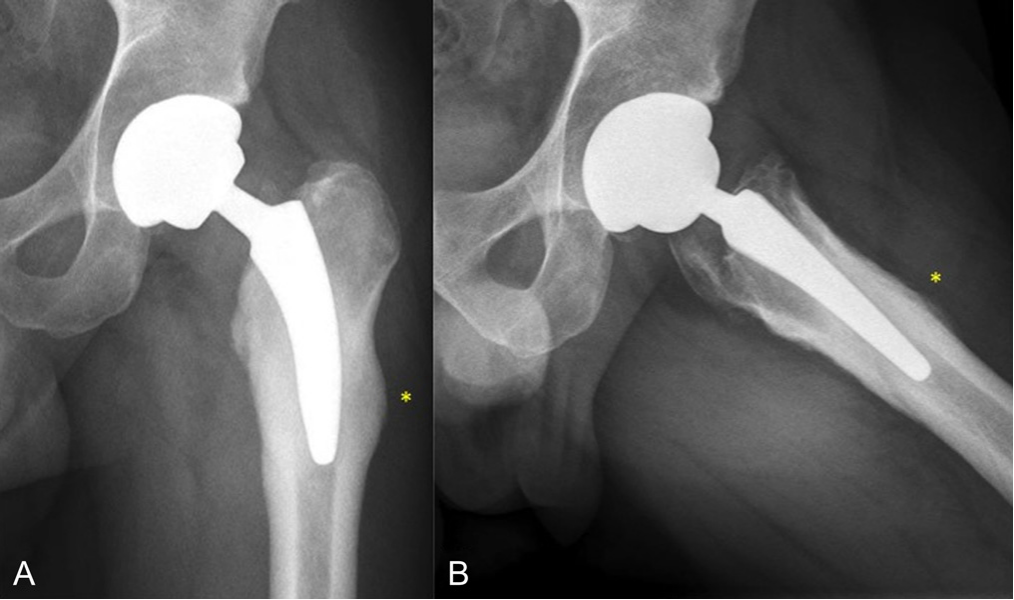
A and B. Left hip's anteroposterior and lateral radiographs at 2 and half years of follow-up, evidencing a hypertrophic callus (*) with no signs of stem loosening, being the patient completely asymptomatic.

A revision arthroplasty was firstly scheduled at the 9th month of follow-up. Since the patient expressed a VAS for pain of 9 points, crutches were indicated until the date of revision. However, pain, which seemed initially unremitting, started to decrease progressively after the biopsy was developed and the patient commenced to protect weight-bearing, until it disappeared at 1-year follow-up. Therefore, a new surgical procedure was dismissed. At 2 and half years of follow-up, the patient remained asymptomatic, with a mHHS of 98 points and playing golf at the same level as before surgery. At final follow-up, radiographs evidenced a healed fracture with a hypertrophic callus at the lateral femoral cortical (Figure 5).

## Discussion

Short-stem THA has already demonstrated excellent mid- and long-term clinical and radiological outcomes [16,17]. However, not all short stem designs are similar in size and shape; [9] thus, distinctive load distributions onto the proximal femur may trigger different patterns of bone remodelling, generating different clinical and radiological results [2]. When thigh pain arises, understanding its potential aetiologies is critical for selecting the right treatment modality. Originally, modern cementless stem designs were attempted to decrease structural rigidity; [4] nonetheless, excessive stress transfer arisen from a mismatch in bending stiffness has been a concern in terms of mechanical alterations at the proximal femur's modulus of elasticity, being pain generation a potential consequence[3]. With the advent of short stems, many articles have focused their attention on thigh pain and plenty of fresher theories have appeared to explain its genesis [2].

After analysing 217 hips operated with a cementless short titanium taper stem, Amendola et al. evidenced that around 23% of patients suffered from thigh pain (with 9% describing moderate to severe pain) at an average of 3-year follow-up [1]. Although finding mild stress shielding in 64% of cases and varus alignment in 8%, the authors could not correlate them statistically with pain or risk of revision. Actually, the authors stated that thigh pain seen in their study might have been related to a modulus mismatch between the stem tip and bone at the high stress subtrochanteric area [1]. Cinotti et al. reported excellent clinical results with scarce bone remodelling at a minimum 9-year follow-up in 72 hips that underwent THA with a short stem with pure metaphyseal fixation (IPS™, DePuy, Varsaw, United States); still, thigh pain was reported by five patients (8%), of which 3 underwent L4 anaesthetic nerve block injection with complete temporal pain relief, whereas 2 had no clear source of pain, which persisted at final follow-up [18]. At a minimum 11-year follow-up, Kim et al. found no cases of thigh pain with the same THA (IPS™, DePuy, Varsaw, United States) used in 630 hips [17]. Ender et al. described unsatisfactory clinical and radiological mid-term outcomes with the CUT™ stem (ESKA Implants, Lübeck, Germany), reporting an 11% revision rate (13/123 cases), of which 2.5% (3 cases) were due to unbearable thigh pain [19]. The authors correlated pain with non-harmonic load distribution onto the proximal femur generating radiographic demineralization in the medial cortical (zone 5) and in the trochanteric area (zone 1) [19]. As far as we interpreted, none of these reports have described a stress fracture as the source of thigh pain. However, it seems difficult to acknowledge whether some of these patients with relentless pain were developing an undiagnosed stress fracture.

Following short-stem THA, postoperative radiographs may sometimes evidence cortical hypertrophy as a consequence of bone remodelling, which is almost always an asymptomatic event. Maier et al. have analysed the clinical and radiological results of their first 100 consecutive short-stem THA with the Fitmore™ stem (Zimmer, Warsaw, Indiana, United States) [20]. After a mean follow-up of 3.3 years (range, 2–4.4 years), survival was 100% with revision for any reason as the endpoint, finding no femoral component loosening. However, cortical hypertrophy was seen on 50 hips, predominantly in Gruen's zones 3 and 5 [13]. Of them, 2 patients reported moderate thigh pain that exacerbated during physical exercise. Pipino et al. also reported a distal cortical hypertrophy rate as high as 48%, mainly in Gruen zones 2, 3 and 5 [21]. In this long-term follow-up study, the authors found thigh pain in 14% of cases, which spontaneously resolved within 1 year. Both authors [20,21] alleged that thigh pain may be a manifestation of temporary instability of the stem, rather than a phenomenon related to tip wedging of the prosthesis. However, we believe that when pain appears in an unbearable fashion, stress fractures must be discarded since bone remodelling might not always explain it, as in the case we reported.

In a critical analysis, Baert et al. expressed that after ruling out loosening, infection or extraarticular sources of thigh pain, other specific causes related to THA constructs should be contemplated [2]. Their bone-preserving nature need femoral preparation to be utterly accurate, considering that rasps should follow the medial femoral curve. As described by their designers, many short stems are supposed to be implanted in slight varus in order to achieve a 3-point contact [22]. Coronal malalignment, particularly in varus position, has been associated with poorer clinical outcomes [23–25]. Seemingly, incorrect stem sizing can also occur more frequently than with conventional stems, producing inadvertent subsidence [26]. In this scenario, micromotion at the bone-prosthesis interface may happen or even periprosthetic fractures might develop, which may be the source of postoperative thigh pain [2]. Although we consider that radiological outcomes were acceptable in the reported case, subtle under-sizing may have remained unaddressed. Nevertheless, whether this issue had been relevant, subsidence should have happened and, since no signs of evident prosthesis sinking or loosening were found, we were not able to correlate such radiological finding with the genesis of a stress fracture.

We found only 3 reports of stress fractures occurring around femoral stems [6–8]. Chun et al. reported 2 cases of insufficiency fractures located at the subtrochanteric area of loose, cemented stems [6]. The authors alleged that osteoporotic bones of elderly patients might produce atraumatic, atypical femoral fractures different to that of the ones reported with bisphosphonate therapy. Bisphosphonates usually require surgery to reach bone healing, whereas in the reported cases teriparatide injection and protected weight-bearing achieved consolidation [27]. Curtin et al. reported 3 similar bisphosphonate-associated fractures with good clinical outcomes after conservative treatment, including alendronate discontinuation [8]. Finally, O'Donnell et al. reported a series of 13 cases of periprosthetic stress fracture around the sleeve-stem junction of the Sivash-Range of Motion™ stem (S-ROM™; DePuy Orthopaedics Inc., Warsaw, Indiana, United States) [7]. Treatment was expectant with resolution of symptoms in all but 3 cases; 1 of which needed revision to a more distally loading stem. The authors concluded that these fractures were caused by weakening of the metaphyseal throat of the anteromedial femoral region by surgical coring and impingement of the distal sleeve at that site, resulting in a stress riser [7]. Therefore, evidence shows that in all of these atypical cases of stress fractures, proximal femoral biomechanics is altered to such an extent that it finally affects the bone interface and produces a cortical crack. In our case, we believe that load was more absorbed by the lateral distal cortical than by the medial calcar, with which contact should be maximal [22]. Hence, as initial contact with the medial calcar was not thorough (Figure 1), excessive loads onto the lateral distal tip may have been implied on the fracture's inception.

We have described an unusual stress fracture at the lateral distal tip of a short cementless stem with metaphyseal fixation. After ruling out infection and loosening, we encourage surgeons to be aware of the existence of periprosthetic stress fractures as a source of thigh pain (sometimes intractable) and despite being infrequent, they should always be contemplated, providing that these cases can be managed conservatively with rest and limited weight-bearing. After this uncommon case, we suggest to align the stem in order to equally distribute loads onto the medial calcar and the lateral femoral cortical [28,29].

## Conflict of interest

The authors declare that they have no conflicts of interest in relation to this article.
